# Comparisons of different general anesthetic techniques on immune function in patients undergoing flap reconstruction for oral cancer

**DOI:** 10.1097/MD.0000000000038653

**Published:** 2024-07-05

**Authors:** Chuanqi Qin, Guo Fan, Lili Huang

**Affiliations:** a State Key Laboratory of Oral & Maxillofacial Reconstruction and Regeneration, Key Laboratory of Oral Biomedicine Ministry of Education, Hubei Key Laboratory of Stomatology, School & Hospital of Stomatology, Wuhan University, Wuhan, Hubei, P.R. China; b Department of Anesthesiology, School and Hospital of Stomatology, Wuhan University, Wuhan, Hubei, P.R. China.

**Keywords:** dexmedetomidine, general anesthesia, immune function, oral cancer, propofol, sevoflurane

## Abstract

**Background:**

Anesthetic-induced immunosuppression is of particular interest in tumor surgery. This study aimed to investigate the influence of the 4 most common general anesthetic techniques on immune function in patients undergoing flap reconstruction for oral cancer.

**Methods:**

116 patients were randomly divided into 4 groups. Patients in group S were given sevoflurane-based anesthesia. Group P was administered propofol-based anesthesia. The SD group received sevoflurane combined with dexmedetomidine anesthesia. The propofol combined with dexmedetomidine anesthesia (PD) group received PD. Blood samples were obtained at 5 time points: baseline (T0), 1 hour after the start of the operation (T1), end of the operation (T2), 24 hours (T3), and 48 hours (T4) after the operation. Lymphocyte subsets (including CD3^+^, CD4^+^, CD8^+^, and B lymphocytes) and dendritic cells were analyzed by flow cytometry. Blood glucose, norepinephrine, and cortisol levels were measured using ELISA and a blood gas analyzer respectively.

**Results:**

In total, 107 patients were included in the final analysis. Immunological indicators, except CD8^+^ counts, were all decreased in groups S, P, and SD at T1-4 compared with the baseline value, and the counts of CD3^+^, CD4^+^, and dendritic cells, as well as CD4^+^/CD8^+^ ratios, were significantly higher in the PD group than in the S, P, and SD at T1-3 (*P* < .05). There were no significant differences between groups P and SD at any observation time point. Intraoperative stress indices, including norepinephrine and cortisol levels, were significantly lower in the PD group than in the other 3 groups at T1-2 (*P* < .05).

**Conclusion:**

These findings suggest that PD as a probably optimal choice can alleviate immunosuppression in patients undergoing flap reconstruction for oral cancer.

## 1. Introduction

In recent years, oral cancer has become a major life-threatening disease in Southeast Asian countries owing to smoking habits, alcohol consumption, and betel quid chewing.^[1]^ Physiologic functions, cosmetic appearance, and psychological well-being may be compromised during treatment with flap reconstruction for oral cancer.^[2]^ According to our previous study, this type of surgery requires approximately 6 to 7 hours.^[3]^ Many studies have shown that inappropriate anesthetic strategies for major surgical manipulation can result in impaired innate and adaptive immunity, leading to consequent tumor recurrence and metastasis.^[4–9]^ In modern society, with the exception of satisfying clinical anesthesia, the maintenance of immune function is also extremely important. Many studies involving anesthesia and immunity have focused on general anesthesia and regional anesthesia^[10,11]^ and few studies have specifically explored the effects of different general anesthetic techniques on immune function in oncology patients.

Sevoflurane, a widely used volatile agent, has gained popularity because of its pharmacodynamic and pharmacokinetic properties.^[12]^ Propofol is a rapid-onset, short-acting sedative and hypnotic agent that is generally used for anesthesia induction and maintenance.^[13]^ Dexmedetomidine, a highly selective α_2_-adrenoceptor agonist, has sedative, anxiolytic, analgesic, and sympatholytic properties.^[14]^ In the present study, we selected the 4 most common combinations of general anesthesia, and the primary aim was to analyze immune cell numbers, including CD3^+^, CD4^+^, CD8^+^, B lymphocytes, and dendritic cells. The secondary aim was to analyze plasma levels of blood glucose, norepinephrine, and cortisol.

## 2. Methods

### 2.1. Ethics committee approval and written informed consent

The present study was approved by the Ethics Committee of the School and Hospital of Stomatology, Wuhan University (IRB2018B23) and registered in the Chinese Clinical Trial Registry (ChiCTR1800018367). This study was conducted at the School and Hospital of Stomatology of Wuhan University, in accordance with the Declaration of Helsinki. Written informed consent was obtained from all the participants prior to the trial.

### 2.2. Patients and study design

Patients classified as American Society of Anesthesiologists physical status I to II and aged 30 to 70 were enrolled. All the patients were scheduled for radical surgery and immediate reconstruction using a forearm flap. None of the patients had a history of endocrine, immune, or major systemic disease. Other exclusion criteria included recent or concurrent chemotherapy and requirement for perioperative blood transfusion. All the surgeries were performed by the same surgeon. A randomization sequence was created by an assistant not involved in the study using computer-generated random numbers, with the group allocation concealed in sequentially numbered opaque envelopes.

### 2.3. Surgical procedure and clinical observations

In the operating room, the right subclavian vein was cannulated for central venous pressure monitoring and the radial artery was cannulated for real-time blood pressure monitoring. Electrocardiography, blood oxygen saturation (SaO_2_), end-tidal carbon dioxide (P_et_CO_2_), and the cerebral state index were continuously monitored during the operation. Anesthesia induction was performed by intravenous injection of etomidate 0.3 mg·kg^−1^, sufentanil 0.4 μg·kg^−1^, and cisatracurium 0.2 mg·kg^−1^ to facilitate nasal tracheal intubation. Anesthesia maintenance included remifentanil 0.2 to 0.3 μg·kg^−1^· min^−1^ and cisatracurium 0.1 mg·kg^−1^·h^−1^. The enrolled patients were randomly divided into group S (inhalation 2%–3% sevoflurane), group P (intravenous infusion 5–10 mg·kg^−1^·h^−1^ propofol), group SD (inhalation 2%–3% sevoflurane and intravenous infusion 0.4 μg·kg^−1^·h^−1^ dexmedetomidine) and group propofol combined with dexmedetomidine anesthesia (PD) (intravenous infusion propofol 5–10 mg·kg^−1^·h^−1^ and dexmedetomidine 0.4 μg·kg^−1^·h^−1^). Mechanical ventilation was performed to maintain P_et_CO_2_ at 35 to 40 mm Hg and SaO_2_ above 98%. The depth of anesthesia was monitored to maintain a cerebral state index between 40 and 60. Thirty minutes before the end of surgery, sufentanil 0.1 μg·kg^−1^ was administered as a loading dose for postoperative analgesia in each patient, and they received the same postoperative intravenous analgesia formula: butorphanol 8 to 10 mg was diluted to 100 mL 0.9% saline with 2 mL h^−1^ constant rate infusion. All patients were transferred to the intensive care unit (ICU) and administered the same treatment. Peripheral venous blood (4 mL) was obtained at 5 time points: baseline (T0), 1 hour after the start of the operation (T1), end of the operation (T2), and 24 hours (T3) and 48 hours (T4) after the operation. Peripheral blood mononuclear cells were isolated by centrifugation using a Lymphoprep (STEMCELL Technologies, CA). Peripheral blood mononuclear cells were then stained with the following antibodies: APC-eFluor 780-conjugated anti-CD45 (HI30), Alexa Fluor 700-conjugated anti-CD3 (UCHT1), FITC-conjugated anti-CD4 (RPA-T4), PE-Cy7-conjugated anti-CD8 (SK1), PC5.5-conjugated anti-CD19 (SJ25C1), APC-conjugated anti-HLA-DR (LN3), and BV421-conjugated anti-CD11C (3.9). All antibodies were obtained from Becton Dickinson, Biolegend or eBioscience. Isotype-matched IgG controls were purchased from eBioscience. The data were analyzed using FlowJo (Tree Star) and gated using side scatter and forward scatter filters. Cortisol and noradrenaline levels were determined by ELISA using a commercial ELISA kit (Cloud-Clone Corp, Co., Ltd., China). Blood glucose was directly detected using a blood gas analyzer (GEM 3500, USA).

### 2.4. Statistical analysis

According to the CD3^+^ counts at T4 in the 4 groups from the preliminary experiment using the Chinese High Intellectualized Statistical Software, with a type-I error of 5% and a power of 80%, 26 was the acceptable patient sample. After anticipating a 10% dropout rate, we recruited a sample size of 29 patients per group. All statistical analyses were performed using the GraphPad Prism 7. Continuous variables were reported as mean and standard deviation. Age, weight, duration of surgery, total intraoperative remifentanil, blood loss, infusion volume, urine volume, ICU observation time, and hospitalization time after surgery were compared using one-way ANOVA. Comparisons of repeated measurement indicators at different observation times in the 4 groups were performed using 2-way ANOVA and Tukey multiple comparison test. Categorical variables are expressed as numbers and percentages (%) and were compared using *chi*-square test and Fisher exact test. Statistical significance was set at *P* < .05.

## 3. Results

### 3.1. Comparisons of general data in the 4 groups

**Figure 1.**
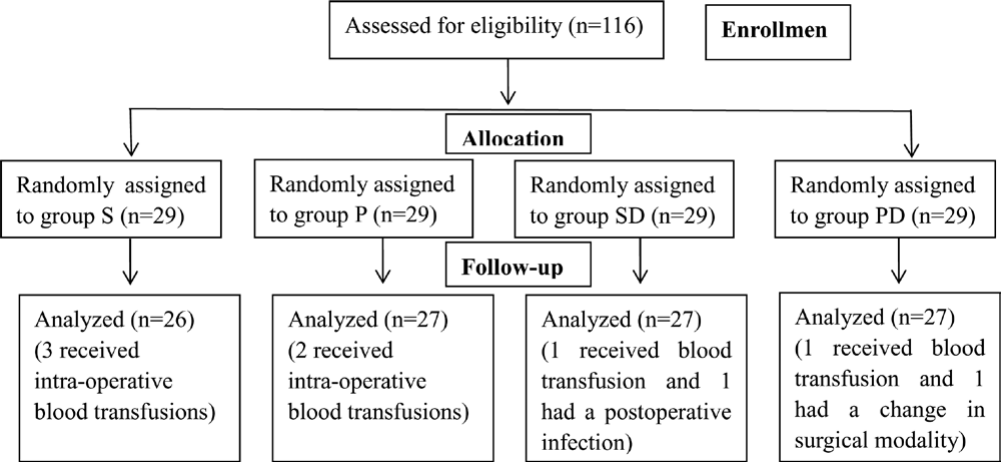
CONSORT flow diagram of the patients recruitment and randomization.

A total of 116 patients who underwent flap reconstruction for oral cancer were recruited. Seven patients were excluded because of blood transfusions, 1 patient had a postoperative infection, and 1 patient had a change in the surgical modality. Therefore, 107 patients were included in the final analysis (Fig. 1). The demographics and surgical profiles of the patients were similar among the 4 groups. There were no significant differences in age, sex, weight, American Society of Anesthesiologists score, duration of surgery, total intraoperative remifentanil, blood loss, infusion, or urine volume (all *P* > .05, Table 1).

**Table 1:** Demographic and surgical profiles of the patients.

	Group S (n = 26)	Group P (n = 27)	Group SD (n = 27)	Group PD (n = 27)	*P* value
Age (y)	52 ± 9	51 ± 8	49 ± 9	51 ± 10	.499
Gender (male/female)	20/6	17/10	22/5	23/4	.233
Weight (kg)	63.5 ± 9.1	63.7 ± 11.7	64.5 ± 9.3	67.0 ± 11.8	.598
ASA (Ⅰ/Ⅱ)	18/8	16/11	19/8	18/9	.825
Duration of surgery (h)	6.6 ± 1.2	6.5 ± 1.0	6.6 ± 0.5	6.1 ± 1.1	.245
Total intraoperative remifentanil (mg)	3.5 ± 0.7	3.4 ± 0.5	3.2 ± 0.7	3.2 ± 0.6	.319
Blood loss (mL)	306 ± 50	300 ± 54	307 ± 53	278 ± 51	.139
Infusion volume (mL)	3350 ± 732	3237 ± 463	3056 ± 307	3030 ± 557	.100
Urine volume (mL)	1319 ± 531	1233 ± 403	1056 ± 276	1152 ± 424	.123

Data shown as mean ± standard deviation or number.

ASA = American Society of Anesthesiologists; P = propofol; PD = propofol combined with dexmedetomidine anesthesia; S = sevoflurane; SD = sevoflurane combined with dexmedetomidine anesthesia.

### 3.2. Comparisons of lymphocyte subsets and dendritic cells in the 4 groups

**Figure 2.**
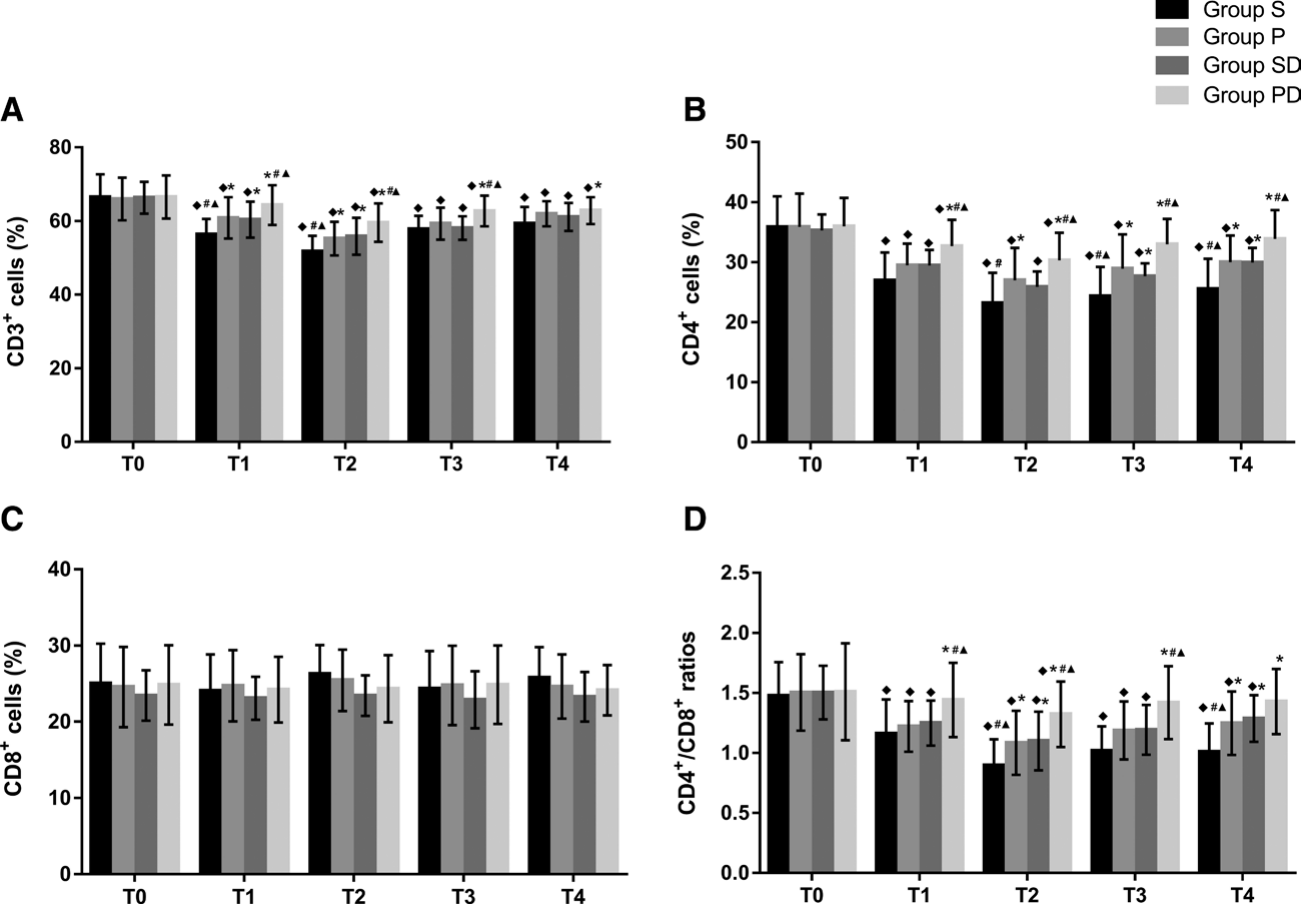
Comparisons of T lymphocyte subsets at different time-points in the 4 groups. (A) Comparisons of CD3^+^ cells in the 4 groups; (B) Comparisons of CD4^+^ cells in the 4 groups; (C) Comparisons of CD8^+^ cells in the 4 groups; (D) Comparisons of CD4^+^ /CD8^+^ ratios in the 4 groups. Compared with T0, ^◆^*P* < .05; compared with group S, ^*^P < .05; compared with group P, ^#^*P* < .05; compared with group SD, ^▲^*P* < .05. D = dexmedetomidine; P = propofol; S = sevoflurane; T0 = baseline; T1 = 1 hour after the start of operation; T2 = end of the operation; T3 = 24 hours after operation and T4 = 48 hours after operation.

**Figure 3.**
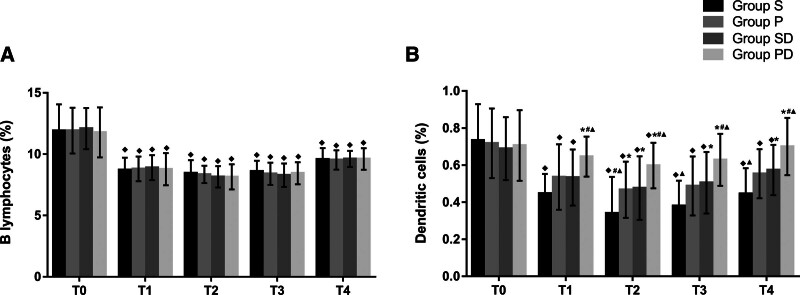
(A) Comparisons of B lymphocytes at different time-points in the 4 groups. Compared with T0, ^◆^*P* < .05. (B) Comparisons of dendritic cells at different time-points in the 4 groups. Compared with T0, ^◆^*P* < .05; compared with group S, ^*^P < .05; compared with group P, ^#^*P* < .05; compared with group SD, ^▲^*P* < .05. D = dexmedetomidine; P = propofol; S = sevoflurane; T0 = baseline; T1 = 1 hour after the start of operation; T2 = end of the operation; T3 = 24 hours after operation and T4 = 48 hours after operation.

The counts of CD3^+^ and CD4^+^cells and the CD4^+^/CD8^+^ ratios decreased in groups S, P, and SD at T1-4 compared with the baseline value, and they were significantly higher in the PD group than in the other 3 groups at T1-3 (*P* < .05, Fig. 2A, B, D). There were no significant differences between groups P and SD at any observation time point. CD8^+^ counts were not significantly different among the 4 groups at T0-4 (*P* > .05, Fig. 2C). The B lymphocyte counts were significantly lower at T1-4 than those at T0 in the 4 groups (*P* < .05, Fig. 3A), but there were no statistically significant differences between the 4 groups at the same time points. In addition, dendritic cell counts were significantly higher in the PD group than in the S, P, and SD at T1-4 (Fig. 3B). These results indicate that patient immunity was suppressed in the 4 groups after anesthesia and surgery, and PD may have been associated with less impairment of cell-mediated innate and adaptive immune responses.

### 3.3. Comparisons of stress indices in the 4 groups

**Figure 4.**
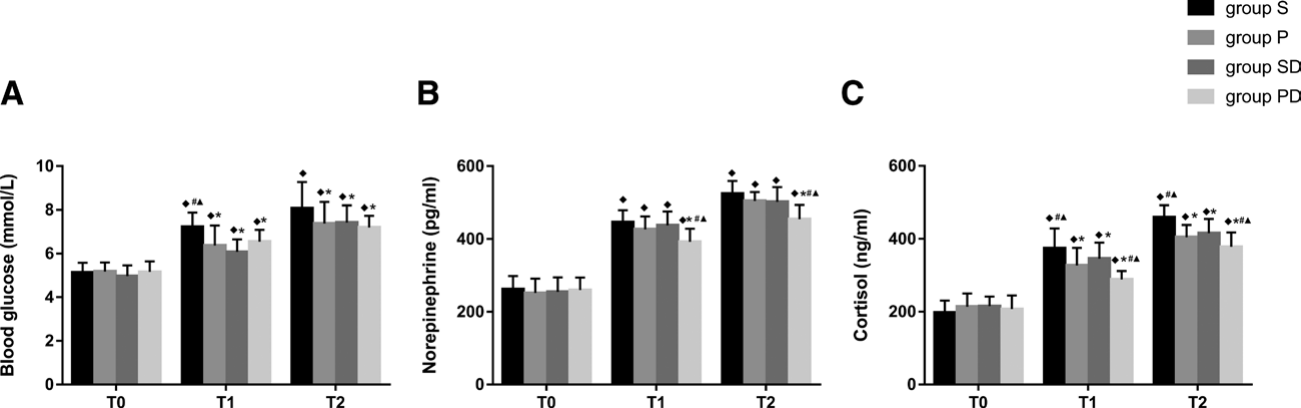
(A) Comparisons of blood glucose at different time-points in the 4 groups; (B) Comparisons of norepinephrine at different time-points in the 4 groups; (C) Comparisons of cortisol at different time-points in the 4 groups. Compared with T0, ^◆^*P* < .05; compared with group S, ^*^P < .05; compared with group P, ^#^*P* < .05; compared with group SD, ^▲^*P* < .05. D = dexmedetomidine; P = propofol; S = sevoflurane; T0 = baseline; T1 = 1 hour after the start of operation; T2 = end of the operation; T3 = 24 hours after operation and T4 = 48 hours after operation.

Compared with T0, the levels of blood glucose, norepinephrine, and cortisol at T1-2 were significantly higher in all 4 groups (*P* < .05, Fig. 4A–C), and the concentrations of norepinephrine and cortisol in the PD group were significantly lower than those in the other 3 groups at T1-2 (*P* < .05, Fig. 4B, C). These results suggest that PD could significantly reduce the intraoperative stress response in patients undergoing flap reconstruction for oral cancer.

### 3.4. Comparisons of recovery parameters and adverse events in the 4 groups

There was no significant difference in ICU observation or hospitalization time after surgery among the 4 groups (*P* = .669 and *P* = .063, respectively). Moreover, no differences were found in the incidence of delirium, flap crisis, or respiratory infections (*P* = .572, 0.270, and 0.556, respectively; Table 2).

**Table 2:** Recovery parameters and complications in the 4 groups.

	Group S (n = 26)	Group P (n = 27)	Group SD (n = 27)	Group PD (n = 27)	*P* value
ICU observation time (h)	41.3 ± 6.1	39.5 ± 5.4	38.6 ± 6.7	39.0 ± 12.9	.669
Hospitalization time after surgery (d)	16.5 ± 3.4	15.8 ± 4.1	14.3 ± 2.9	14.6 ± 2.6	.063
Delirium	0	1	1	0	.572
Flap crisis	2	1	0	0	.270
Respiratory infection	1	1	0	0	.556

Data shown as mean ± standard deviation or number.

ICU = intensive care unit; P = propofol; PD = propofol combined with dexmedetomidine anesthesia; S = sevoflurane; SD = sevoflurane combined with dexmedetomidine anesthesia.

## 4. Discussion

Our results indicated that the patients’ immune function was suppressed after anesthesia and surgery in all 4 groups, and PD alleviated the decrease in CD3^+^, CD4^+^, and dendritic cell counts and CD4^+^/CD8^+^ ratios, and reduced the concentrations of norepinephrine and cortisol in patients undergoing flap reconstruction for oral cancer. These findings are consistent with those of previous studies^[3,15–17]^ which demonstrated that sevoflurane could directly inhibit natural killer cells and induce apoptosis of T lymphocytes, negatively affecting the immune system.^[15,16]^ Propofol has been shown to have protective effects on immunity and direct anticancer effects.^[8,9,17]^ Our previous study showed that dexmedetomidine had an inhibitory effect on the reduction of immune cell populations in patients undergoing oral cancer surgery.^[3]^

Despite the development of chemotherapy, radiotherapy, and new hormonal and immunological treatments, it is estimated that more than 52% of patients with tumors worldwide will require surgical resection between 2018 and 2040.^[18]^ Oral cancer is one of the most common malignancies in Southeast Asia with an increasing impact on young people.^[2,19]^ In order to repair function and appearance, surgical resection and immediate flap reconstruction are the mainstays of treatment for oral cancer patients.^[3]^ Cancer cells are often found in circulation, hidden in distant organs through micrometastases, or dislodged from tumors during excision.^[4–6]^ Surgical-induced and anesthetic-induced immunosuppression have been implicated in the significant effects on tumor growth and recurrence.^[4–9]^ Liu et al found that the most significant immune suppression comes from the induction of anesthesia rather than surgery.^[20]^ At present, the impact of general anesthetics on immune function is a new area of interest.

The immune system is divided into innate and adaptive components.^[5]^ Dendritic cells are professional antigen-presenting cells that are considered critical factors in cell-mediated innate immunity.^[21]^ Peripheral T and B lymphocytes are key mediators of the adaptive immune response, and conventional T lymphocytes are composed of CD4^+^ and CD8^+^ T cells. The reduction in immune cells and CD4^+^/CD8^+^ ratio are closely related to postoperative recovery and cancer metastasis. Although there is no literature reporting the relationship between the degree of reduction in immune cells and clinical effects, their significant decline usually indicates an unfavorable prognosis.^[3,5]^ In the present study, we found that PD alleviated the decrease in the counts of CD3^+^, CD4^+^, and dendritic cells, as well as the CD4^+^/CD8^+^ ratios, compared with those in the other 3 groups (*P* < .05 at T1-3, Figs. 2A, B, D, and 3B). The results implied that this anesthetic technique is associated with an improvement in the cellular innate and adaptive immune responses and may be beneficial for anti-tumor therapy. Our results are consistent with those of the previous studies. Longhini et al revealed that propofol can be associated with improved recurrence-free and overall survival in patients undergoing oncologic surgery,^[9]^ and this anesthetic may be a promising immunoregulatory agent for tumor treatment.^[17]^ Kun et al found that the application of dexmedetomidine can reduce the secretion of inflammatory factors and decrease the inhibition of immunity in patients undergoing radical surgery for colon carcinoma.^[22]^ Another study by Wang et al reported that dexmedetomidine can attenuate perioperative stress and inflammation and protect the immune function of surgical patients.^[23]^

In addition to the direct influence of anesthetics on immunity, the immune system is also affected by the surgical stress response, which is characterized by activation of the hypothalamus-pituitary-adrenal axis and the sympathetic nervous system.^[24]^ Although the stress response improves the chances of survival following injury, it may be detrimental in modern surgical practice. Increasing evidence suggests that surgical stress alters the tumor microenvironment, facilitates an immunosuppressive state for approximately 2 weeks, and results in impaired recruitment and function of crucial immune cells.^[4,25]^ The fundamental purpose of anesthesia is to inhibit the stress response associated with surgery. The present study showed that PD decreased the levels of norepinephrine and cortisol more than the other 3 methods (*P* < .05 at T1-2, Fig. 4B, C). These results suggest that propofol and dexmedetomidine can significantly reduce the stress response during flap reconstruction surgery. We speculate that the lower reduction in immune cell populations in the PD group may be related to a more suppressed stress response.

Previous studies have reported that opioids, which are commonly used as analgesic agents in surgery, have immunosuppressive effects.^[26,27]^ Therefore, in the present study, we ensured that the management of pain in each group was similar, there was no significant difference in terms of the amount of perioperative remifentanil (*P* = .319, Table 1), and the patients received the same postoperative intravenous analgesia. In addition, although there was no significant difference in the recovery parameters and adverse events among the 4 groups, a retrospective cohort study found that propofol anesthesia for colon cancer surgery was associated with better survival irrespective of the tumor-node-metastasis stage.^[7]^ And dexmedetomdine was associated with a lower 30-day mortality ratio in cardiac surgery and a low incidence of delirium in ICU patients.^[28,29]^ Therefore, we need to extend the observation time and sample size in future studies.

Our study has several limitations. First, although the short-term clinical consequences were analyzed, the long-term effects of different general anesthetic techniques require further study. Additionally, owing to the physiological regularity of hormone secretion and susceptibility to interference,^[24]^ we selected the observation time from baseline to the end of surgery for stress levels. Finally, we analyzed the changes in critical immune cells. According to Relland et al, immune function measures are based on antigen presentation, cytokine production capacity, and relative proportions of cell populations;^[30]^ therefore, detection at the molecular and immunoendocrine levels is required in the future.

## 5. Conclusion

Although anesthetic technology is close to maturity, there is no gold standard for cancer surgery. Our findings indicate, for the first time, that PD alleviated the decrease in the counts of CD3^+^, CD4^+^, dendritic cells, and CD4^+^/CD8^+^ ratios, as well as reduced the concentrations of norepinephrine and cortisol. This general anesthetic technique, as a highly probable beneficial choice, can attenuate immunosuppression in patients undergoing flap reconstruction for oral cancer.

## Acknowledgments

The authors thank all colleagues of the Department of Anesthesiology, School, and Hospital of Stomatology, Wuhan University.

## Author contributions

**Conceptualization:** Chuanqi Qin, Lili Huang.

**Data curation:** Chuanqi Qin, Lili Huang.

**Investigation:** Guo Fan.

**Methodology:** Guo Fan.

**Resources:** Lili Huang.

**Writing – original draft:** Chuanqi Qin, Lili Huang.

**Writing – review & editing:** Chuanqi Qin, Lili Huang.
